# Utility of silver birch and house dust mite extracts derived from licensed sublingual tablets for nasal allergen challenge

**DOI:** 10.1002/clt2.12360

**Published:** 2024-05-23

**Authors:** Bianca Olivieri, Ana Jimenez Gil, Kostadin Stoenchev, Stephen R. Durham, Guy Scadding

**Affiliations:** ^1^ Asthma, Allergy and Clinical Immunology Section University Hospital of Verona Verona Italy; ^2^ Department of Allergy Royal Brompton & Harefield Hospitals NHS Trust London UK; ^3^ Allergy and Clinical Immunology National Heart and Lung Institute Imperial College London London UK

**Keywords:** allergen immunotherapy, allergic rhinitis, birch pollen, house dust mite, nasal allergen challenge

## Abstract

**Background:**

Nasal allergen challenge (NAC) is used to investigate the effects of allergen exposure and assess treatment efficacy in allergic rhinitis (AR). This study aims to establish dose‐responses to NAC using licensed silver birch (SB) pollen and house dust mite (HDM) sublingual tablets as sources of the allergen extracts in participants with AR.

**Methods:**

Sixteen volunteers with HDM‐induced perennial AR and 15 volunteers with SB pollen‐induced seasonal rhinitis underwent a graded up‐dosing NAC with extracts derived from HDM allergen (Acarizax®) and SB (Itulazax®) tablets, respectively. Total nasal symptom score (TNSS, range 0–12) and peak nasal inspiratory flow (PNIF) were recorded before, at 10 min and at the end of the NAC. The dose of each allergen that provoked a TNSS of at least 7 (“provoking dose 7”) in most allergic participants was identified. NACs using the “provoking dose 7” were performed on 5 non‐allergic individuals to test for irritant effects. The “provoking dose 7” of HDM extract was used in a subgroup of two SB allergic, non‐HDM allergic, volunteers, and vice versa for SB extract, to test for allergen specificity of the responses.

**Results:**

Most patients experienced a TNSS of at least 7/12 at a median concentration of 1500 AU/mL for both SB pollen and HDM. The average decline in PNIF at this dose was 63.15% for SB and 63.99% for HDM. NACs using the 1500 AU/mL concentrations were performed on 5 non‐allergic individuals with no symptomatic or PNIF response. 1500 AU/mL of HDM extract produced no symptoms in SB allergics nor 1500 AU/mL SB extract in HDM allergics.

**Conclusion:**

For both SB and HDM extracts, the optimal allergen dose for NAC to cause a moderate‐severity response (“provoking dose 7/12”) was 1500 AU/mL. Licensed sublingual allergen tablets provide a readily available and inexpensive source of SB and HDM extracts for use in future interventional studies in AR.

## 1 INTRODUCTION

Nasal Allergen Challenge (NAC) is a useful tool in the investigation of allergic rhinitis (AR) reproducing an allergic reaction of the nose under standardized and controlled conditions.^1^, ^2^ NAC is used to explore both the clinical and immunological responses to allergen exposure,^3^, ^4^, ^5^ and it is also applied as a surrogate outcome to evaluate the efficacy of treatments for AR, such as allergen immunotherapy.^6^ In a clinical setting, NAC can be helpful to further clarify the diagnosis of rhinitis, especially in patients with a discrepancy between symptoms and allergy tests.^7^ The procedure has been shown to be safe, with patients generally demonstrating good tolerance to the process.^8^

The efficacy of NAC as a valid tool is critically dependent on the standardization of allergen extracts in terms of their concentration and stability. However, their availability is restricted to certain regions.^2^ A previous study addressed this gap by comparing the effects of two different grass pollen allergen extracts in NAC. It demonstrated that a lyophilized pollen tablet, intended for sublingual immunotherapy (SLIT), was a viable alternative to a traditional dry powder allergen.^9^

SLIT freeze‐dried, lyophilized tablets are designed to quickly disperse under the tongue, ensuring the rapid and complete delivery of allergens to the immune‐competent cells within the oral mucosa. This process is critical for activating the immune system and developing tolerance towards specific allergens. These formulations have been consistently shown to release the full content of allergens rapidly, in as little as 15–30 s. Studies comparing freeze‐dried with compressed SLIT tablet formulations highlight the superior efficiency of freeze‐dried tablets in allergen release. They not only disintegrate faster but also achieve full recovery of the allergen content in a soluble form, facilitating a more efficient sublingual allergen delivery.^10^, ^11^, ^12^

Standardization remains an unmet need for house dust mite (HDM) and silver birch (SB) pollen NAC. The aim of the study is to evaluate the dose‐responses to NAC using SB pollen and HDM allergen extracts in participants with AR using licensed sublingual tablets. This could have significant implications for the standardization of NAC and its use in future interventional studies involving these two common allergens.

## 2 METHODS

### 2.1 Study population

Twenty‐nine volunteers with moderate to severe HDM and/or SB pollen induced AR were recruited at the allergy clinic at the Royal Brompton Hospital, London. Individuals allergic to both allergens were eligible for both parts of the study. Five nonatopic individuals were also recruited. Participants gave written, informed consent; the study was approved by the regional ethics committee (ref. 22/WM/0166) and registered with clinicaltrials.gov (NCT05378594).

Inclusion criteria were age 18–65 years, a history of perennial rhinitis for at least 2 years (for HDM) or a history of seasonal AR in February‐May for at least 2 seasons (for SB) and positive skin prick test (≥5 mm wheal diameter) to HDM and/or SB extract (ALK‐Abello, Denmark). All participants were non‐active smokers, defined as <10 pack‐years and none in the last 6 months. Exclusion criteria were history of previous allergen immunotherapy to the relevant allergen, FEV1 (forced expiratory volume in 1 s) < 70% predicted at screening, perennial asthma requiring regular inhaled corticosteroids, history of emergency visit or hospital admission for asthma in the previous 12 months, chronic obstructive pulmonary disease, chronic or recurrent acute sinusitis, history of life‐threatening anaphylaxis or angioedema, and ongoing systemic immunosuppressive treatment. Participants had not used antihistamines for at least 5 days and corticosteroids or other antiallergy medications for at least 2 weeks prior to the NAC. The study was conducted outside the SB pollen season in the United Kingdom.

### 2.2 Study design

HDM allergic volunteers underwent a graded up‐dosing NAC with HDM allergen tablet extract in normal saline; SB allergic volunteers underwent a graded up‐dosing NAC with SB allergen tablet extract in normal saline; dual allergic individuals were given the option of undergoing challenges to both allergens, at least 4 weeks apart.

Baseline Peak Expiratory Flow Rate (PEF),^13^ Peak Nasal Inspiratory Flow (PNIF),^14^, ^15^ Total Nasal Symptom Score (TNSS)^16^, ^17^ and sneeze count were recorded. The NAC was initiated only if the baseline TNSS was 2 or less. TNSS, PNIF and sneeze count were then recorded after each increasing dose of allergen administered during the NAC. The TNSS is a 12‐point scale with 4 categories: sneezing, nose running, nose blockage, and itching, each rated from 0 to 3. Since a NAC is considered positive, ranging from moderate to strong, based on an increase in the TNSS from the baseline by 3–5 points–excluding patients who have a baseline TNSS of more than 2–and considering an increase of at least 5 to indicate a clearly positive response to NAC, we have identified a cutoff of 7 (“provoking dose 7”) to classify patients with at least a moderate response to the NAC.^2^, ^3^, ^18^

The primary endpoint was the area under the curve (AUC) for TNSS during NAC with each allergen. Secondary endpoints include AUC for change from baseline PNIF and sneeze count during NAC, the median allergen dose required to give a TNSS of 7/12 (“provoking dose 7”), peak TNSS score, peak PNIF fall%, and the peak sneeze count.

Following the above, we conducted NAC using the established “provoking dose 7” for each allergen on five nonatopic individuals to confirm the absence of any irritant effects. The protocol for the nonatopic group entailed a two‐step NAC with Itulazax® and Acarizax®, administered 30 min apart. The challenge sequence was as follows: 0 AU/mL (saline control 1), 0 AU/mL (saline control 2), and then the established “provoking dose 7” for HDM, then for SB or vice versa.

**FIGURE 1:**
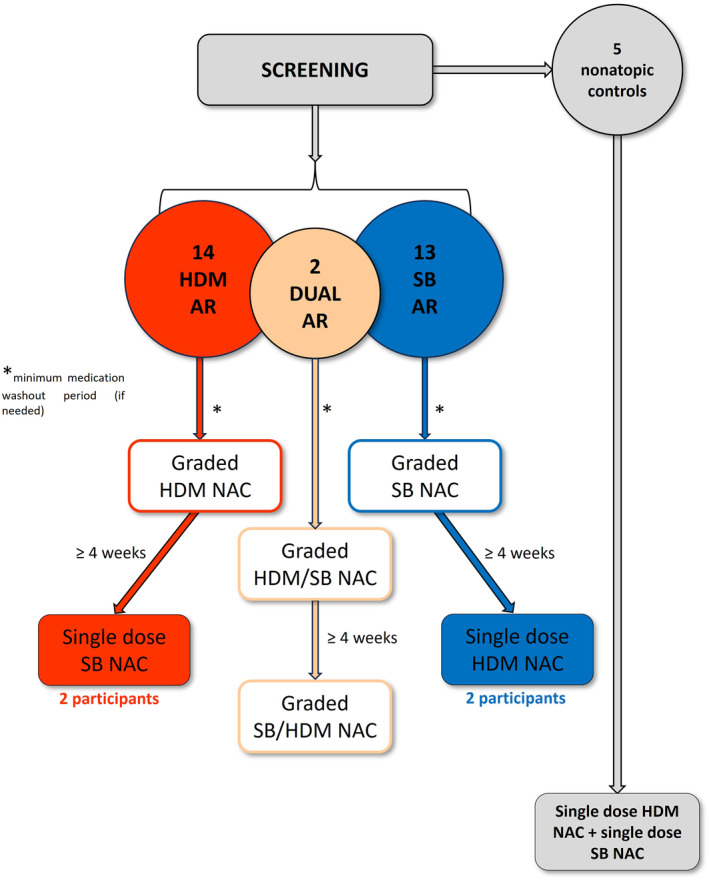
Flowchart of the study design. This diagram illustrates the screening and subsequent NAC protocol for 29 volunteers with AR. Participants were divided into groups based on their allergen reactivity: 14 were allergic to HDM, 13 to SB, and 2 were dual‐allergic (dual AR) to both HDM and SB. Five nonatopic controls were also included. The flowchart details the NAC procedure, starting with graded challenges for HDM and SB in order to find the “provoking dose 7,” followed by a single‐dose challenge with the “provoking dose 7” of SB in a subgroup of two participants allergic to HDM, and vice versa with the “provoking dose 7” of HDM in two participants allergic to SB. The two dual‐allergic participants first underwent a graded NAC with one allergen, for instance SB, followed by a graded NAC with the other allergen, such as HDM, after a minimum interval of 4 weeks. The five non‐atopic individuals underwent a single‐dose NAC using the provoking dose 7 for both allergens. AR, allergic rhinitis; HDM, house dust mite; NAC, Nasal allergen challenge; SB, silver birch.

We also tested the “provoking dose 7” of HDM extract in a subgroup of 2 SB allergic, non‐HDM allergic, volunteers, and vice versa, with the “provoking dose 7” of SB (or HDM) extract to demonstrate allergen specificity of responses. A minimum gap of 4 weeks was required between this NAC and the first NAC. Figure 1 shows the flow chart illustrating the design of the study.

### 2.3 Nasal allergen challenge

The allergen extracts used were Itulazax® tablets (SB pollen allergen sublingual lyophilized tablets, ALK‐Abello, Denmark) and Acarizax® tablets (HDM allergen sublingual lyophilized tablets, ALK‐Abello, Denmark). The allergen extracts used in this study are licensed in the UK and EU for sublingual administration in the treatment of AR.

Serial dilutions were prepared starting from two dissolved tablets of either Acarizax 12 SQ‐HDM or Itulazax 12 SQ‐SB in 1.5 mL of saline solution. This stock solution had an allergen concentration of 16 SQ/mL for both allergens. For ease of subsequent dilution calculations, this solution was assigned a value of 30,000 arbitrary units (AU/mL). Five graduated dilutions were then prepared as follows: 10,000 AU/mL corresponding to 5.33 SQ/mL, 5000 AU/mL to 2.66 SQ/mL, 1500 AU/mL to 0.8 SQ/mL, 500 AU/mL to 0.26 SQ/mL, and 100 AU/mL to 0.052 SQ/mL (see Supporting Information S1: Appendices).

The NAC procedure involved administering a single spray (100 μL) of each dilution into both nostrils of the participant using a Bi‐dose nasal applicator device (Aptar Pharma, Germany). TNSS and PNIF were recorded prior to intervention and then 10 min after challenge with each dose at the following concentrations: 0 AU/mL (saline control 1); 0 AU/mL (saline control 2); 100 AU/mL; 500 AU/mL; 1500 AU/mL; 5000 AU/mL; 10,000 AU/mL, with 15 min interval between doses until the top concentration of 10,000 AU/mL regardless of TNSS score, unless the participant experienced bothersome symptoms and preferred not to continue. At the end, observations were repeated, including PEFR. When required, a single tablet of desloratadine 5 mg or similar was administered.

### 2.4 Statistical analyses

The mean AUC for TNSS, change from baseline PNIF and sneeze count were calculated for each allergen NAC (HDM, SB). Missing values were imputed. Comparisons of AUC values between different allergens and between allergen and control challenges–including challenges to non‐sensitized allergens and those in nonatopic individuals–were analyzed using Student's *t*‐test. *p*‐values of <0.05 were considered statistically significant. The median allergen dose first producing a TNSS of at least 7 was recorded for each allergen. The peak TNSS score and peak fall in PNIF were calculated for each individual and the mean and standard error were calculated for each allergen. The peak sneeze count was calculated in the same way. Microsoft Excel was used for data organization and preliminary graphing. NumPy and SciPy libraries within the Python programming language were employed to calculate AUC for the TNSS, PNIF and sneeze count and Spearman's correlations. No power calculation was performed as this was an exploratory study for HDM and SB. The sample size is comparable to that used in our previous studies on different allergens.^3^, ^4^, ^5^, ^9^

## 3 RESULTS

### 3.1 Main demographic and clinical characteristics of the study population

We enrolled 29 volunteers, of which 14 were diagnosed with AR induced by HDM, 13 were affected by SB‐induced AR, and 2 were allergic to both allergens. The control group consisted of five healthy volunteers with a mean age of 45.4 years, predominantly female (M:F = 1:4). In whom sensitization to inhalant allergens at SPT and seasonal asthma were absent. Spirometry confirmed FEV1 >80% predicted in all participants (mean 102.6% predicted). Controls were all non‐smokers. Demographic and clinical characteristics of the study population and controls are presented in Table 1.

**TABLE 1:** Demographical and clinical characteristics of patients according to allergen sensitization.

Demographic and clinical characteristics	HDM (16)	SB (15)	Healthy controls (5)
Age (mean)	36.87	38.13	45.40
Gender (M:F)	1:1	2:3	1:4
Allergic rhinitis duration (years mean)	19.25	15.57	0
SPT (MM mean)	6.65	5.86	0
Seasonal asthma (*n*.)	3	4	0
FEV1 (%)	93.68	97.40	102.6%
Active smoker (*n*.)	0	0	0

### 3.2 Total nasal symptom score

**FIGURE 2:**
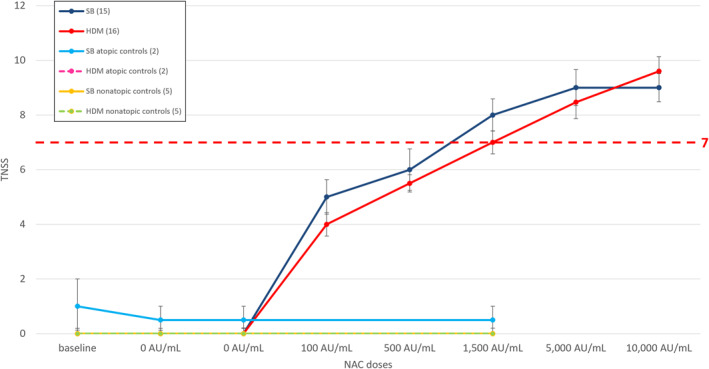
Median TNSS and standard errors during NAC. The dark blue line represents the NAC with SB allergen, the red line corresponds to HDM, and the yellow and dashed green lines represent nonatopic controls for SB and HDM, respectively. Atopic controls exposed to HDM are represented by the dashed pink line, which overlaps with the yellow and dashed green lines of the nonatopic controls and is therefore not visible graphically. Atopic controls exposed to SB are illustrated by the light blue line. The dashed red line indicates the cutoff score of seven (provoking dose 7). HDM, house dust mite; NAC, Nasal allergen challenge; SB, silver birch; TNSS, total nasal symptom score.

All the 16 participants allergic to HDM completed the graded up‐dosing NAC with HDM allergen tablet, except one who did not reach the last two doses because of reported mild dyspnea, but no fall in peak flow was detected; all 15 SB‐allergic volunteers completed all doses of NAC with SB. The two volunteers allergic to both allergens underwent a NAC with each allergen, separated by an interval of at least 4 weeks. For all allergic subjects, median TNSS increased with each subsequent dose following the initial two saline solutions in the NAC challenge for both allergens. Most patients experienced a TNSS of at least 7 out of 12, called the “provoking dose 7,” at a median concentration of 1500 AU/mL for HDM. The provoking dose 7 was calculated at a median concentration of 1500 AU/mL also for SB pollen. Five nonatopic individuals underwent the NAC with the “provoking‐dose‐7” of SB and then of HDM without having any effects on TNSS. The “provoking dose 7” of HDM extract was tested in a subgroup of two SB allergic, non‐HDM allergic, volunteers without having any effects on TNSS. Vice versa, a subgroup of two HDM allergic, non‐SB allergic, volunteers were exposed to the “provoking dose 7” of SB extract, with a median TNSS of 0.5 (Figure 2). See Table S1 for median and IQR for TNSS for each dose and group.

Mean AUC for TNSS for each allergen NAC was calculated and no statistically significant difference between SB and HDM allergens was found (*p* = 0.59). When compared to the control groups, both the SB and HDM NACs showed a significant difference in TNSS AUC (*p* < 0.001 for both comparisons) (see Figure S1).

The mean peak TNSS was 10.2 (standard error 0.43) for HDM NAC and 10.0 (standard error 0.49) for SB NAC. For the non‐atopic controls, the mean peak was calculated at 0.2 for both HDM and SB NACs. For the atopic SB allergic participants exposed to NAC with HDM, the mean peak TNSS was 0; for the atopic HDM allergic participants exposed to NAC with SB, the mean peak TNSS was 1.

### 3.3 Peak nasal inspiratory flow

**FIGURE 3:**
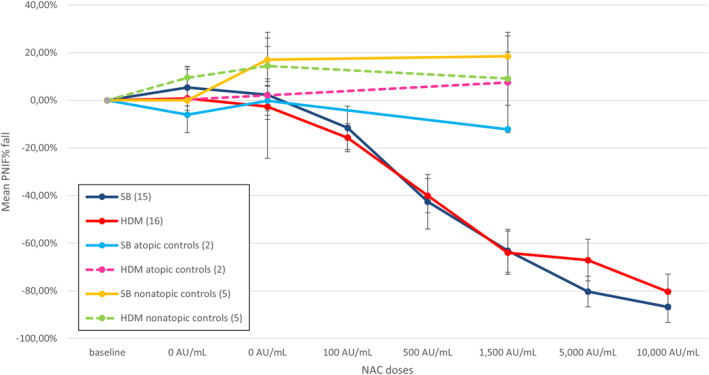
The mean percentage fall in peak nasal inspiratory flow (PNIF) and standard errors during NAC. The dark blue line represents the NAC with SB allergen, the red line corresponds to HDM, and the yellow and dashed green lines represent nonatopic controls for SB and HDM, respectively. Atopic controls exposed to HDM are depicted by the dashed pink line, atopic controls exposed to SB are illustrated by the light blue line. HDM, house dust mite; NAC, Nasal allergen challenge; SB, silver birch.

The mean baseline PNIF, with standard deviation, was 111.33 ± 40 L/min for SB and 112.81 ± 43.78 L/min for HDM allergic participants. Non‐atopic controls exhibited a mean baseline PNIF of 110.00 ± 30.82 L/min. For atopic controls exposed to SB and HDM, the mean baseline PNIFs were 165 ± 7.07 L/min and 127.50 ± 81.32 L/min, respectively. The mean percentage fall in PNIF during NAC with SB and HDM allergens was calculated for each dose. The average decline in PNIF at the “provoking dose 7” was 63.15% for SB NAC and 63.99% for HDM NAC in allergic subjects. In the nonatopic group, there was no PNIF fall during the NACs with SB and HDM allergens but an increase of 18.47% and 9.12%, respectively. An increase of 7.53% in PNIF was also observed in the subgroup of SB allergic volunteers exposed to HDM NAC. In the subgroup of HDM allergic volunteers exposed to SB NAC, a 12.13% fall in PNIF was observed (Figure 3). See Table S2 and S3 for PNIF mean, standard deviation and standard error for each dose and group.

Mean PNIF fall AUC for each allergen NAC was calculated and no statistically significant difference between SB and HDM allergens was found (*p* = 0.48). When compared to the control groups, both the SB and HDM NACs showed a significant difference in mean PNIF fall AUC with *p* < 0.001 for the non‐atopic control challenges and *p* = 0.025 and *p* = 0.022 for the atopic HDM and SB challenges, respectively (see Figure S2).

The mean peak PNIF% fall was 73.48% (standard error 7.67%) for HDM NAC and 88.38% (standard error 5.80%) for SB NAC, with no significant difference between groups (*p* = 0.13).

A Spearman's correlation was run to examine the correlation between TNSS and PNIF% fall during the nasal challenges. There was a strong, negative correlation, statistically significant for both HDM (*r* −0.71, *p* < 0.001) and SB (*r* −0.80, *p* < 0.001). There were also significant inverse correlations between nasal congestion score alone and PNIF% fall for both allergens, HDM (*r* −0.72, *p* < 0.001) and SB (*r* −0.86, *p* < 0.001) (See Figures S3 and S4).

### 3.4 Sneeze count

**FIGURE 4:**
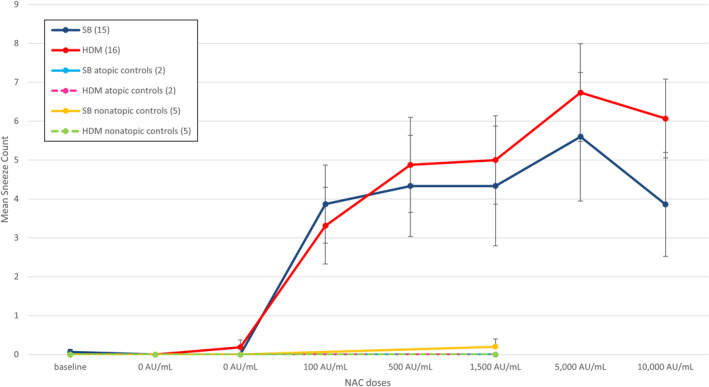
Mean sneeze count and standard errors for SB (blue) and HDM (red). Yellow line represents nonatopic SB controls, dashed green line represents nonatopic HDM controls. Atopic controls exposed to HDM (dashed pink line) and SB (light blue line) exhibit curves that overlap with those of the non‐atopic HDM controls, showing values equal to zero. HDM, house dust mite; SB, silver birch.

The mean sneeze count during NAC with SB and HDM allergens was calculated for each dose. The sneeze count reached a peak at 5000 AU/mL, followed by a fall thereafter. The mean sneeze count at the “provoking dose 7” was 4.33 (standard error 1.54) for SB NAC and 5.00 (standard error 1.14) for HDM NAC in allergic subjects. The mean sneeze count at the “provoking dose 7” was 0.00 for nonatopic volunteers who underwent NAC with HDM and 0.20 for nonatopic volunteers who underwent NAC with SB. No sneezes were recorded in the subgroup of SB allergic volunteers exposed to HDM NAC and in the subgroup of HDM allergic volunteers exposed to SB NAC (Figure 4).

Sneeze count AUC for each allergen NAC was calculated and no statistically significant difference between SB and HDM allergens was found (*p* = 0.58). When compared to the nonatopic group, HDM allergic patients showed a significant difference in sneeze count AUC (*p* = 0.003) for NAC with HDM. No significant difference in the sneeze count AUC was observed for patients allergic to SB during the NAC with SB allergen when compared to the nonatopic control group (*p* = 0.06). It should be noted that within the SB allergic patient group, there was an outlier with one individual not sneezing during the NAC with SB allergen, despite all other parameters (TNSS and PNIF) confirming the allergic response and aligning with those of the other allergic patients. This anomaly, along with a single sneezing event observed in the nonatopic group during the NAC, rendered the comparison of mean sneeze counts between groups statistically non‐significant.

The mean peak sneeze count was 8.68 (standard error 1.12) for HDM NAC and 7.66 (standard error 1.56) for SB NAC. For the non‐atopic group, no sneezes were recorded during HDM NAC; the mean sneeze count peak was 0.2 for the nonatopic group during SB NAC. The mean sneeze count peak was 0 for HDM allergic participants during NAC with SB and for SB allergic participants during NAC with HDM.

During the study, no serious or unexpected adverse events were reported. One participant allergic to HDM and with a history of seasonal asthma developed mild breathlessness without a fall in PEF at a dose of 1500 AU/mL. The participant received 200 mcg inhaled salbutamol with resolution of symptoms.

## 4 DISCUSSION

This study demonstrates a dose‐responsive, reproducible, and safe approach to NAC using solubilized HDM and SB pollen tablets. We were able to determine the “provoking dose 7” for each allergen, which elicited moderate AR symptoms in all allergic participants. Neither HDM nor SB NACs provoked a response in non‐atopic individuals, confirming that the allergen extracts are non‐irritating. Furthermore, participants allergic to HDM showed no allergic response to the SB NAC and vice versa, proving the specificity of the used allergen extracts. NACs with these allergens were safe, with no severe or unexpected adverse events reported. This study provides the standardization of NAC procedures using allergen extracts from licensed sublingual tablets for HDM and SB pollen.

One limitation of the study is the modest sample size although comparable to other studies.^3^, ^4^, ^9^ Despite this, the study maintained adequate statistical power. In addition, the absence of previous NAC data among participants precludes insights into their responses to traditional extracts. A further limitation relates to the allergen units used; the allergen concentrations are expressed in Arbitrary Units (AU/mL) due to the challenges associated with obtaining precise information on the major allergen content in sublingual allergen immunotherapy products. Commercial allergen products have different concentration units depending on the manufacturer and this prevents direct comparison of allergen concentrations.^2^ Nevertheless, the equivalent concentration in μg/mL is reported (see Supporting Information S1: Appendices) as published for the NAC with HDM using Acarizax^19^ and the methodological rigor in preparing the NAC doses and the evaluation of symptom responses through the TNSS have allowed for a reliable measurement of the biological potency of the allergen extracts employed. Further studies are needed in particular to assess allergen thresholds in relation to clinical symptoms in order to assess whether the procedure may also be of value to assist diagnosis in clinical and research settings.

The 2018 European Academy of Allergy and Clinical Immunology (EAACI) position paper^2^ emphasizes the unmet needs in NAC methodologies, such as standardization of allergen dose and quality, application techniques, and the necessity of a titration process. Recent studies have explored the NAC method for HDM using Allerkin® HDM extract,^20^ and for SB pollen, comparing the outcomes of NAC with Allergopharma® allergoid diluted SB and those of environmental exposure chambers.^21^ This study contributes to this field by proposing a standardized protocol for HDM and SB pollen. The lack of availability of commercial aqueous allergen extracts is increasingly a problem, and prior research has already demonstrated that sublingual tablets could serve as a viable alternative for NAC with grass pollen.^9^ This study extends the validation to HDM and SB licensed lyophilizate sublingual tablets which are extensively used for allergen immunotherapy in clinical settings. The findings of this study, along with those from the previous investigation on grass pollen, support the use of sublingual tablets as reliable sources of allergens for NACs involving grass, SB, and HDM.

NAC with HDM and SB pollen allergens is both easy to administer and reproducible, making the method suitable for application in research settings. Outcomes are effectively monitored using clinical scores such as the TNSS and objective parameters such as the PNIF. This protocol could be applied to future clinical trials assessing the efficacy and action mechanisms of anti‐allergic drugs and allergen immunotherapy for AR caused by HDM or SB pollen. In a clinical setting, it could also serve to verify allergen reactivity in polysensitized patients before initiating allergen immunotherapy or to more accurately diagnose rhinitis in cases where symptoms and allergy test results are incongruent.

In conclusion, this study has determined an allergen dosage that elicits a moderate‐severity response–designated as the “provoking dose 7”–in individuals with HDM or SB pollen induced AR. By utilizing licensed sublingual tablets, we have confirmed that these allergen sources are appropriate for conducting NAC with both SB pollen and HDM extracts. The findings validate the use of these allergen extracts in a clinical setting and establish a foundation for future interventional and experimental studies.

## AUTHOR CONTRIBUTIONS

Guy Scadding and Stephen R. Durham conceived the idea for the study and wrote the protocol. Bianca Olivieri and Ana Jimenez Gil recruited participants and undertook the study with assistance from Guy Scadding and Kostadin Stoenchev. Bianca Olivieri wrote the manuscript with input from Ana Jimenez Gil, Guy Scadding and Stephen R. Durham. Ana Jimenez Gil, Kostadin Stoenchev, Stephen R. Durham, and Guy Scadding critically revised the manuscript. The final version was revised and approved by all authors.

## CONFLICT OF INTEREST STATEMENT

GS has received honoraria from ALK Abello for lecturing and participation in a modified Delphi advisory panel. SRD has received honoraria for consultancies from ALK, Revelo and Regeneron and honoraria for lectures from Abbott laboratories, ALK, PneumoUpate GmbH, Stallergenes, and Tori.

## Supporting information

Supporting Information S1
