# Supplementary material for: Utility of silver birch and house dust mite extracts derived from licensed sublingual tablets for nasal allergen challenge
Source: Clin Transl Allergy. 2024 May 23;14(5):e12360. doi: 10.1002/clt2.12360 (PMC11112400; doi:10.1002/clt2.12360)
Supplement: Supplementary file 1 — Supporting Information S1 [file CLT2-14-e12360-s001.docx]

**Appendices**

**Preparation of solutions and Nasal Spray for NAC with tablet allergen extract (Itulazax® /Acarizax®)**. Major allergen concentrations derived from published data (Acarizax) and from assumption of similar major allergen concentration to Grazax tablets (Itulazax).

| **Itulazax® (SB)** | | | |
| --- | --- | --- | --- |
| **Tablet** | **SQ** | **AU** | **μg** |
| 1 tablet | 12 |  | 15 |
| 2 tablets | 24 |  | 30 |
| **Tablet** | **SQ/ml** | **AU/ml** | **μg/ml** |
| STOCK | 16 | 30 000 AU/ml | 20 |
|  | 5.33 | 10 000 AU/ml | 6.66 |
|  | 2.66 | 5 000 AU/ml | 3.33 |
|  | 0.8 | 1500 AU/ml | 1 |
|  | 0.26 | 500 AU/ml | 0.33 |
|  | 0.052 | 100 AU/ml | 0.066 |

| **Acarizax® (HDM)** | | | |
| --- | --- | --- | --- |
| **Tablet** | **SQ** | **AU** | **μg** |
| 1 tablet | 12 |  | 30 |
| 2 tablets | 24 |  | 60 |
| **Tablet** | **SQ/ml** | **AU/ml** | **μg/ml** |
| STOCK | 16 | 30 000 AU/ml | 40 |
|  | 5.33 | 10 000 AU/ml | 13.33 |
|  | 2.66 | 5 000 AU/ml | 6.67 |
|  | 0.8 | 1500 AU/ml | 2 |
|  | 0.26 | 500 AU/ml | 0.67 |
|  | 0.052 | 100 AU/ml | 0.13 |

The preparation of the stock solution involved two 12-SQT tablets to be placed into a sterile specimen container (5 or 7mL Bijoux tube); 1.5mL of sterile 0.9% saline was added, dropwise, using a 1 mL syringe, onto the tablets, ensuring that the tablets are completely dissolved; the solution was shaken gently to aid mixing. This created a stock solution at a concentration of 16 SQT/mL. For ease of subsequent dilution calculations, this solution was assigned a value of 30,000 arbitrary units (AU/mL). Serial dilutions were made using normal saline as above, following the steps described:

a. 10,000 AU/mL: add 0.5 mL of stock solution (30,000 AU/mL) to 1.0 mL normal saline in a sterile specimen container, mix gently;

b. 5,000 AU/mL: add 0.5 mL of 10,000 AU/mL solution to 0.5 mL normal saline in a sterile specimen container, mix gently;

c. 1,500 AU/mL: add 0.2 mL of stock solution (30,000 A U/mL) to 3.8 mL normal saline in a sterile specimen container, mix gently;

d. 500 AU/mL: add 0.2 mL of 5,000 AU/mL solution to 1.8 mL normal saline in a sterile specimen container, mix gently;

e. 100 AU/mL: add 0.2 mL of 5,000 AU/mL solution to 0.8 mL normal saline in a sterile specimen container, mix gently.

The serial dilutions were made no more than 2 hours prior to the start of each NAC.

The nasal spray device preparation involved adding 2 x 115 µl of diluted allergen (100 - 500 – 1,500 – 5,000 – 10,000 AU/ml) or saline into a bidose glass vial using a 200 µl pipette. Then, it was inserted a rubber bung using a non-disposable bidose device. Finally, it was assembled a single disposable bidose device containing the prepared glass vial.

Single stock solution was made each day of study NAC visits and disposed after visit completion.

**Supplementary Table 1.** Median and Interquartile Range (IQR) for TNSS for each allergen dose and for each group.

|  | **SB allergic** | | **Non Atopic Control SB** | | **Atopic Control SB** | |
| --- | --- | --- | --- | --- | --- | --- |
|  | **Median** | **IQR** | **Median** | **IQR** | **Median** | **IQR** |
| **baseline** | 0 | 1 | 0 | 0 | 1 | 1 |
| **0 AU/mL** | 0 | 0,5 | 0 | 0 | 0,5 | 0,5 |
| **0 AU/mL** | 0 | 1 | 0 | 0 | 0,5 | 0,5 |
| **100 AU/mL** | 5 | 4 |  |  |  |  |
| **500 AU/mL** | 6 | 4,5 |  |  |  |  |
| **1,500 AU/mL** | 8 | 4 | 0 | 0 | 0,5 | 0,5 |
| **5,000 AU/mL** | 9 | 3,5 |  |  |  |  |
| **10,000 AU/mL** | 9 | 2 |  |  |  |  |
|  | **HDM allergic** | | **Non Atopic Control HDM** | | **Atopic Control HDM** | |
|  | **Median** | **IQR** | **Median** | **IQR** | **Median** | **IQR** |
| **baseline** | 0 | 1 | 0 | 0 | 0 | 0 |
| **0 AU/mL** | 0 | 1 | 0 | 0 | 0 | 0 |
| **0 AU/mL** | 0 | 1 | 0 | 0 | 0 | 0 |
| **100 AU/mL** | 4 | 1,5 |  |  |  |  |
| **500 AU/mL** | 5,5 | 1 |  |  |  |  |
| **1,500 AU/mL** | 7 | 2 | 0 | 0 | 0 | 0 |
| **5,000 AU/mL** | 8,5 | 3,25 |  |  |  |  |
| **10,000 AU/mL** | 9,6 | 4 |  |  |  |  |

**Supplementary Table 2.** PNIF% mean, standard deviation and standard error for each allergen dose and for each group.

|  | **SB allergic** | | | **Non Atopic Control SB** | | | **Atopic Control SB** | | |
| --- | --- | --- | --- | --- | --- | --- | --- | --- | --- |
|  | **Mean** | **St.Dev.** | **St.Err.** | **Mean** | **St.Dev.** | **St.Err.** | **Mean** | **St.Dev.** | **St.Err.** |
| **baseline** | 0,00% | 0,00% | 0,00% | 0,00% | 0,00% | 0,00% | 0,00% | 0,00% | 0,00% |
| **0 AU/mL** | 5,39% | 29,88% | 7,71% | 0,00% | 0,00% | 0,00% | -6,07% | 0,26% | 0,18% |
| **0 AU/mL** | 2,30% | 25,97% | 6,71% | 17,04% | 20,45% | 9,15% | -0,18% | 8,58% | 6,07% |
| **100 AU/mL** | -11,57% | 35,28% | 9,11% |  |  |  |  |  |  |
| **500 AU/mL** | -42,56% | 44,31% | 11,44% |  |  |  |  |  |  |
| **1,500 AU/mL** | -63,15% | 34,91% | 9,01% | 18,47% | 19,17% | 8,58% | -12,13% | 0,52% | 0,37% |
| **5,000 AU/mL** | -80,27% | 25,08% | 6,48% |  |  |  |  |  |  |
| **10,000 AU/mL** | -86,72% | 25,54% | 6,59% |  |  |  |  |  |  |
|  | **HDM allergic** | | | **Non Atopic Control HDM** | | | **Atopic Control HDM** | | |
|  | **Mean** | **St.Dev.** | **St.Err.** | **Mean** | **St.Dev.** | **St.Err.** | **Mean** | **St.Dev.** | **St.Err.** |
| **baseline** | 0,00% | 0,00% | 0,00% | 0,00% | 0,00% | 0,00% | 0,00% | 0,00% | 0,00% |
| **0 AU/mL** | 0,81% | 20,10% | 5,02% | 9,51% | 10,29% | 4,60% | 0,39% | 19,66% | 13,90% |
| **0 AU/mL** | -2,64% | 21,58% | 5,39% | 14,44% | 18,29% | 8,18% | 2,12% | 37,40% | 26,45% |
| **100 AU/mL** | -15,71% | 23,54% | 5,88% |  |  |  |  |  |  |
| **500 AU/mL** | -40,06% | 28,78% | 7,20% |  |  |  |  |  |  |
| **1,500 AU/mL** | -63,99% | 36,10% | 9,02% | 9,12% | 25,04% | 11,20% | 7,53% | 29,76% | 21,04% |
| **5,000 AU/mL** | -67,09% | 34,93% | 8,73% |  |  |  |  |  |  |
| **10,000 AU/mL** | -80,37% | 29,57% | 7,39% |  |  |  |  |  |  |

**Supplementary Table 3.** PNIF mean, standard deviation and standard error for each allergen dose and for each group.

|  | **SB allergic** | | | **Non Atopic Control SB** | | | **Atopic Control SB** | | |
| --- | --- | --- | --- | --- | --- | --- | --- | --- | --- |
|  | **Mean** | **St.Dev.** | **St.Err.** | **Mean** | **St.Dev.** | **St.Err.** | **Mean** | **St.Dev.** | **St.Err.** |
| **baseline** | 111,33 | 40,77 | 10,53 | 110,00 | 30,82 | 13,78 | 165,00 | 7,07 | 5,00 |
| **0 AU/mL** | 113,93 | 39,69 | 10,25 | 124,00 | 18,17 | 8,12 | 155,00 | 7,07 | 5,00 |
| **0 AU/mL** | 110,00 | 39,01 | 10,07 | 126,00 | 20,74 | 9,27 | 165,00 | 21,21 | 15,00 |
| **100 AU/mL** | 96,00 | 41,58 | 10,74 |  |  |  |  |  |  |
| **500 AU/mL** | 64,67 | 47,60 | 12,29 |  |  |  |  |  |  |
| **1,500 AU/mL** | 39,00 | 36,61 | 9,45 | 124,00 | 18,17 | 8,12 | 145,00 | 7,07 | 5,00 |
| **5,000 AU/mL** | 21,00 | 24,22 | 6,25 |  |  |  |  |  |  |
| **10,000 AU/mL** | 11,43 | 19,94 | 5,15 |  |  |  |  |  |  |
|  | **HDM allergic** | | | **Non Atopic Control HDM** | | | **Atopic Control HDM** | | |
|  | **Mean** | **St.Dev.** | **St.Err.** | **Mean** | **St.Dev.** | **St.Err.** | **Mean** | **St.Dev.** | **St.Err.** |
| **baseline** | 112,19 | 43,13 | 10,78 | 110,00 | 30,82 | 13,78 | 127,50 | 81,32 | 57,50 |
| **0 AU/mL** | 112,19 | 42,66 | 10,67 | 120,00 | 31,62 | 14,14 | 120,00 | 56,57 | 40,00 |
| **0 AU/mL** | 110,94 | 49,64 | 12,41 | 122,00 | 21,68 | 9,70 | 115,00 | 35,36 | 25,00 |
| **100 AU/mL** | 94,38 | 42,73 | 10,68 |  |  |  |  |  |  |
| **500 AU/mL** | 71,88 | 48,06 | 12,01 |  |  |  |  |  |  |
| **1,500 AU/mL** | 47,19 | 49,60 | 12,40 | 114,00 | 11,40 | 5,10 | 125,00 | 49,50 | 35,00 |
| **5,000 AU/mL** | 37,00 | 42,42 | 10,60 |  |  |  |  |  |  |
| **10,000 AU/mL** | 24,00 | 38,37 | 9,59 |  |  |  |  |  |  |


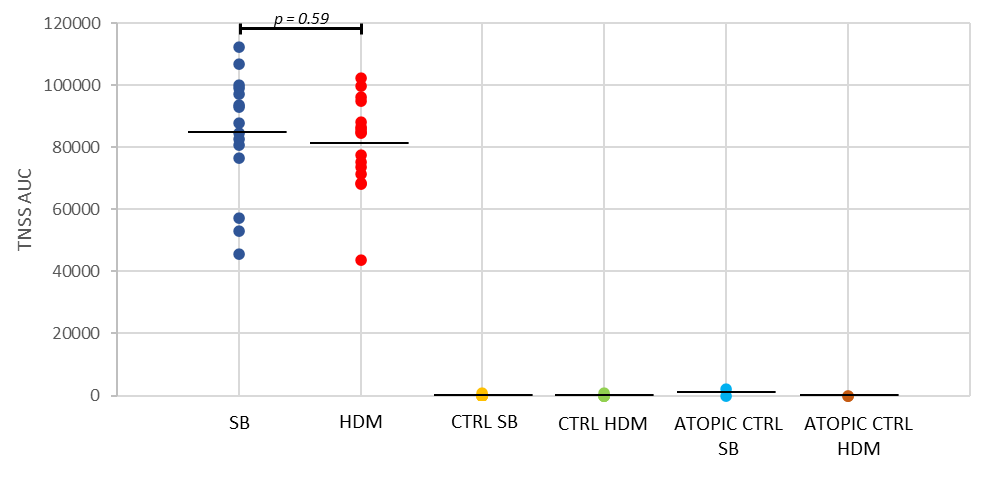


**Supplementary Figure 1.** Mean TNSS AUC, individual values, paired *t* test.


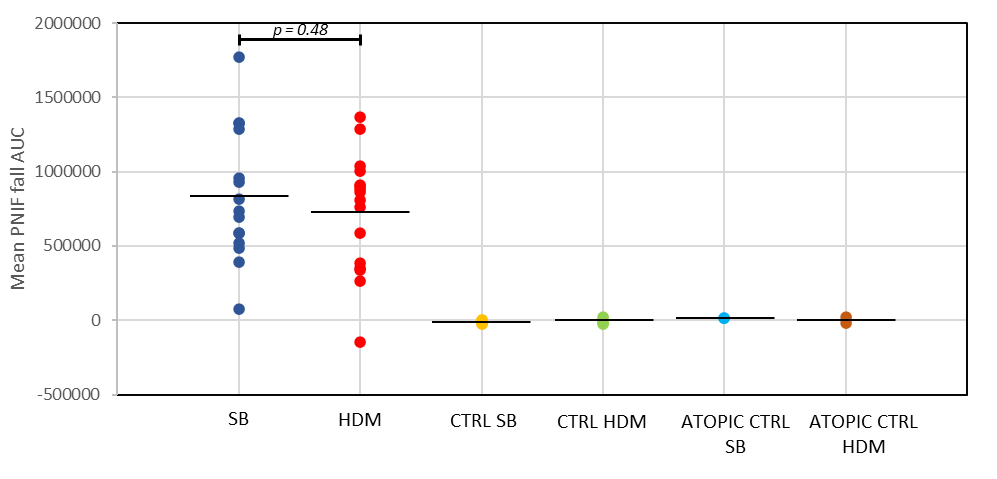


**Supplementary Figure 2.** Mean PNIF fall AUC, individual values, paired *t* test


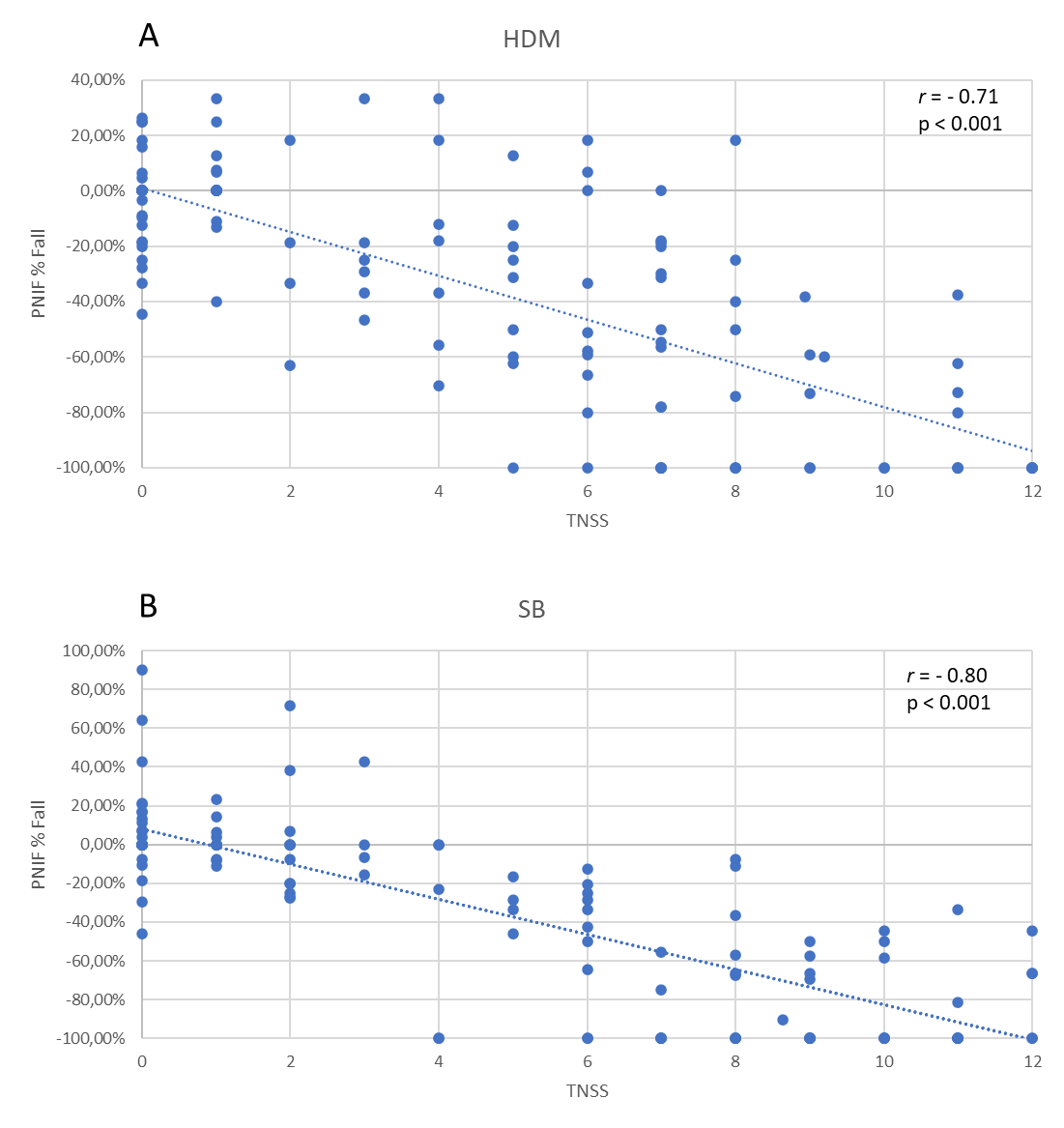


**Supplementary Figure 3.** Spearman's correlation between TNSS and PNIF% fall during the nasal challenges with HDM (A) and SB (B).

**
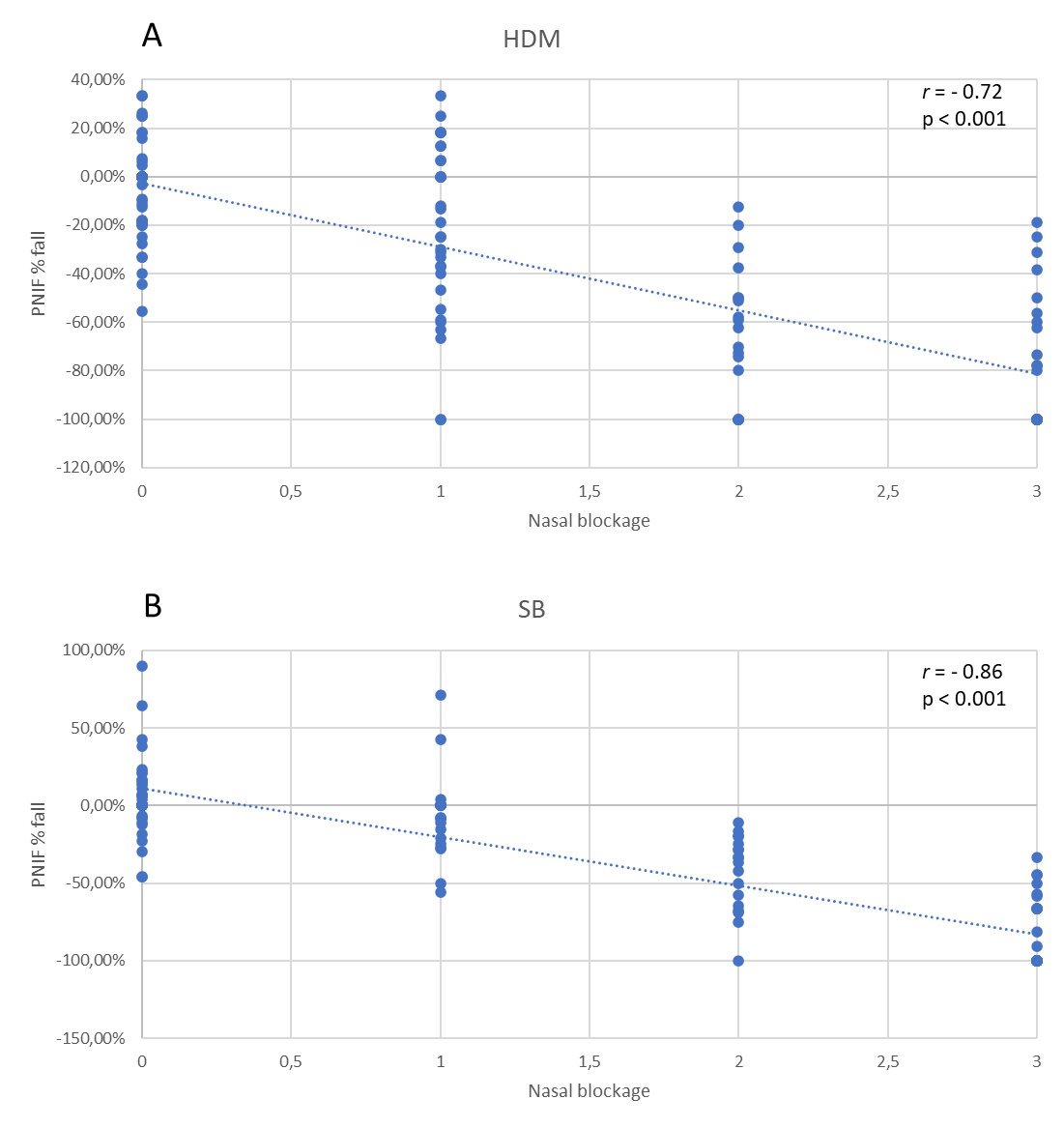
**

**Supplementary Figure 4.** Spearman's correlation between nasal blockage and PNIF % fall during the nasal challenges with HDM (A) and SB (B).
